# EFNA3 as a predictor of clinical prognosis and immune checkpoint therapy efficacy in patients with lung adenocarcinoma

**DOI:** 10.1186/s12935-021-02226-x

**Published:** 2021-10-13

**Authors:** Mingming Deng, Run Tong, Zhe Zhang, Tao Wang, Chaonan Liang, Xiaoming Zhou, Gang Hou

**Affiliations:** 1 Department of Pulmonary and Critical Care Medicine, Center of Respiratory Medicine, China-Japan Friendship Hospital, Beijing, 100029 China; 2 Graduate School of Peking Union Medical College, Chinese Academy of Medical Sciences, Peking Union Medical College, Beijing, 100029 China; 3 National Center for Respiratory Medicine, Beijing, 100029 China; 4 Institute of Respiratory Medicine, Chinese Academy of Medical Sciences, Beijing, 100029 China; 5 National Clinical Research Center for Respiratory Diseases, Beijing, 100029 China; 6 Department of Pathology, Shengjing Hospital of China Medical University, Shenyang, 110001 China; 7 Department of Pathology, Shenyang KingMed Center for Clinical Laboratory Co., Ltd., Shenyang, 110001 China; 8 Department of Pulmonary and Critical Care Medicine, First Hospital of China Medical University, Shenyang, 110001 China; 9 Department of Pulmonary and Critical Care Medicine, Fourth Hospital of China Medical University, Shenyang, 110001 China

**Keywords:** EFNA3, Lung cancer, Prognosis, Biomarker, Immunotherapy response

## Abstract

**Background:**

Ephrin receptors (Eph) and their ligands, called ephrins, function in various disease processes. However, the expression level and prognostic value of Eph/ephrins in lung adenocarcinoma (LUAD) are still unclear.

**Methods:**

The Oncomine and GEPIA databases were used to explore the differential expression of Eph/ephrins in LUAD. Kaplan–Meier plotter was selected to explore the prognostic value of Eph/ephrins. The cBioPortal database was used to analyze the genetic variation of the EFNA3 gene. Immunohistochemistry was used to analyze the expression level and clinical value of ephrin-A3 protein in clinical LUAD tissue. Weighted coexpression network analysis (WGCNA) and gene set enrichment analysis (GSEA) identified the potential regulatory mechanism of EFNA3. CCK-8 assays and colony-forming experiments were used to investigate whether EFNA3 can regulate cell proliferation ability in LUAD. Analysis of lactate, ATP, and glucose uptake levels was used to explore the effect of EFNA3 on glycolysis ability. In addition, we investigated the relationship between EFNA3 and tumor infiltrating immune cells (TIICs). Finally, the potential immunotherapy response prediction value of EFNA3 was also explored.

**Results:**

In this study, we found that EFNA3 expression was significantly correlated with both overall survival (OS) and progression-free survival (PFS) in LUAD patients based on a comprehensive analysis of the Eph/Ephrin family. Next, the expression of the EFNA3 protein was increased in LUAD tissues and was designated an independent prognostic risk factor. Mechanistically, EFNA3 may be involved in nuclear division, synaptic function, and ion channel activity-related pathways. In vitro experiments confirmed the role of EFNA3 in promoting LUAD cells and showed that it could regulate glycolytic capacity. Moreover, EFNA3 was negatively associated with immunity, stromal infiltration, and several TIICs. Finally, EFNA3 was found to be positively related to multiple immunotherapy biomarkers.

**Conclusions:**

In conclusion, increased EFNA3 in LUAD patients predicted worse clinical prognosis, promoted LUAD cell proliferation and glycolysis ability, and was related to immunotherapy response.

## Introduction

Lung cancer was the most commonly diagnosed cancer in 2018, accounting for nearly 20% of cancer deaths that year [1]. Lung adenocarcinoma (LUAD) is a predominant pathologic subtype of lung cancer [2]. Despite progress in comprehensive therapies, including surgery, radiotherapy, and targeted therapy, over the past 20 years, the OS of LUAD patients remains poor [3, 4]. Therefore, continued exploration of the prognostic biomarkers in patients and therapeutic targets for LUAD is necessary to provide more individualized therapies that could lead to better prognoses.

Ephrin receptors (Eph) and ephrins, their membrane-anchored ligands, are essential for the development and organization of multicellular organisms. Eph/ephrins have been shown to function in various disease-related processes [5–8]. Ephs are activated by binding to ephrin ligands. Ephs and ephrins are divided into two subfamilies. The first are EphA receptors (EphA1 to EphA8 and EphA10), which primarily bind to GPI-anchored ephrin-A ligands (EFNA1 to EFNA5). The second are the EphB receptors (EphB1 to EphB4 and EphB6) that preferentially engage transmembrane ephrin-B ligands (EFNB1 to EFNB3) [9, 10]. Aberrant expression of Eph/ephrins has been identified in different types of human cancers. The mode of action has been implicated to affect malignant tumors through two-way signal transduction as well as interaction with other signaling systems [11]. Recent studies have shown that EphA2, Ephrin-A1, and Ephrin-B2 are closely implicated in the prognosis of patients with LUAD [12, 13]. However, the expression level and prognostic value of Eph/ephrins in lung adenocarcinoma (LUAD) are still unclear.

In this study, the expression levels and prognostic value of the Eph/ephrins family in LUAD were analyzed using bioinformatics. EFNA3 was further analyzed to verify the expression levels and prognostic value in LUAD through clinical samples. Finally, the biological function and regulatory mechanism of EFNA3 were explored based on bioinformatics analysis and in vitro experiments. Taken together, these findings indicate that increased EFNA3 in LUAD patients predicted worse clinical prognosis, promoted LUAD cell proliferation and glycolysis ability, and was related to the immunotherapy response.

## Materials and me thods

### Bioinformatics analysis

The Oncomine database (http://www.oncomine.org) is a tumor microarray database, that has collected 715 microarray data sets as well as 86,733 cancer and normal tissue sample data sets[14]. In this study, the Oncomine database was used to analyze the expression levels of the Eph/Ephrin family in different types of cancers.

The GEPIA database [15] (http://gepia.cancer-pku.cn/) was also used to analyze Eph/Ephrin expression in the TCGA-LUAD database.

Likewise, the Kaplan–Meier plotter (http://kmplot.com) is a tool for evaluating prognostic markers for breast cancer, ovarian cancer, lung cancer, and gastric cancer [16]. In this study, the Kaplan–Meier plotter was used to analyze the prognostic value of the Eph/Ephrin family in lung adenocarcinoma. The cBioPortal database (http://www.cbioportal.org) is an open-source database of DNA copy number, DNA methylation, and mutations based on the TCGA database [17]. Here, cBioPortal was used to analyze genetic variation of the *EFNA3* gene.

Gene set enrichment analysis (GSEA) was performed as previously described[18]. The *EFNA3*-high/low groups were divided following the median expression of *EFNA3* based on the TCGA database. The MSigDB KEGG gene set was used as a reference.

### Patients and tissue samples

There were 74 primary LUAD tissue samples and paired normal lung tissue samples collected from **S**hengjing Hospital, China Medical University. Pathological staging of patients in this study was done according to the eighth edition of the AJCC TNM staging. 31 patients with stage I, 35 patients with stage II, and 8 patients with stage III–IV. None of these patients had undergone chemotherapy or radiotherapy before surgery. In the eighth edition of the TNM classification, patients with stage I were divided into stage IA (tumor ≤ 3 cm, no lymph node metastasis and no distant metastasis) and stage IB (a tumor > 3 cm but ≤ 4 cm, no lymph node metastasis, and no distant metastasis). In this study, 31 patients with stage I were divided into stage IA (n = 19) and stage IB (n = 12). 12 patients with stage IB and 43 patients with stage II–IV received adjuvant chemotherapy as platinum-based regimens after surgery following clinical treatment guidelines. Due to tissues were collected via surgery, follow-up treatment does not affect the Ephrin-A3 expression. Clinicopathological data were obtained from medical records and pathological reports. The study was approved by the Human Ethics Review Committee of Shengjing Hospital, China Medical University, and written informed consent was obtained from all enrolled patients.

### Immunohistochemistry (IHC) analysis

IHC staining was performed and IHC scores were measured as described previously [18]. Formalin-fixed, paraffin-embedded primary LUAD tissues were sectioned into 4 µm thickness. IHC staining was performed according to the manufacturers’ instructions (UltraSensitiveTM SP; MXB, China). In short, LUAD tissue sections were fixed and blocked using routine laboratory procedures, followed by incubation with primary antibodies of EFNA3 (1:100; Catalog# ab89472; Abcam) overnight at 4 °C. The sections were then incubated with secondary antibody at room temperature, and visualized with 3,3-diamino-benzidine tetrahydrochloride (DAB; Maixin Biotech, China). Each section was evaluated and scored independently by two pathologists. A semi-quantitative scoring system was used in this assay. Intensity was scored as “0” (negative), “1” (weak), “2” (moderate), and “3” (strong). We also calculated the proportion of tumor cells within each category. The proportional score was then multiplied by the staining intensity score to generate a final IHC score. The IHC scores ranged from zero (minimum) to 300 (maximum). Patients with high expression of EFNA3 was defined as detectable immunoreactions with an IHC score > 10.

### Weighted co-expression network analysis (WGCNA)

To explore the potential function of *EFNA3*, weight co-expression network analysis (WGCNA) was constructed. Approximately 4096 genes (according to variance) were extracted to construct WGCNA using a "WGCNA" package. The adjacency matrix was converted into the topological overlap matrix (TOM) when the power of β was equal to 3 (R^2^ = 0.868). Similar modules were merged following a height cutoff of 0.25. The module of highest correlation with *EFNA3* expression was selected to explore its biological function through GO and KEGG analyses.

### Cell culture and transfection

Human bronchial epithelial cell line HBE, the human LUAD cell lines (A549, H1299, PC9, and HCC827) were obtained from the China Infrastructure of Cell Line Resource. All the cell lines were mycoplasma negative and authenticated using short tandem repeat authentication. Cells were cultured in dulbecco's modified eagle medium (DMEM, Gibco, USA) supplemented with 10% fetal bovine serum (FBS, Gibco, USA) at 37 °C in a humidified 5% CO_2_ incubator. Small interfering RNA (siRNA) transfection was performed as previously described [18]. The siRNA sequence of EFNA3 was 5′‐ATCCTCCGGTTCTTGCAGT‐3′. And the siRNA sequence of negative control (NC) was 5′- UUCUCCGAACGUGUCACGUTT-3′. Transfection efficiency was identified at 48 h after transfection by qRT-PCR and western blot analyses.

### RNA isolation and real-time polymerase chain reaction (PCR)

RNA was isolated and reverse transcribed as previously described [19]. The primer sequences for amplification were as follows: forward (5’- AGTTCTCGGAGAAGTTCCAGCG-3’) and reverse (5’- CAGCAGACGAACACCTTCATCC-3’) for *EFNA3* (forward: 5′-CCCGGGGAGGTAGTGACGAAAAAT-3′, reverse: 5′-CGCCCGCCCGCTCCCAAGAT-3′) for the 18S control.

### Plasmid transfections

The overexpression plasmids containing whole coding sequence of EFNA3(NCBI Reference Sequence: NM_004952.5; ATGGCGGCGGCTCCGCTGCTGCTGCTGCTGCTGCTCGTGCCCGTGCCGCTGCTGCCGCTGCTGGCCCAAGGGCCCGGAGGGGCGCTGGGAAACCGGCATGCGGTGTACTGGAACAGCTCCAACCAGCACCTGCGGCGAGAGGGCTACACCGTGCAGGTGAACGTGAACGACTATCTGGATATTTACTGCCCGCACTACAACAGCTCGGGGGTGGGCCCCGGGGCGGGACCGGGGCCCGGAGGCGGGGCAGAGCAGTACGTGCTGTACATGGTGAGCCGCAACGGCTACCGCACCTGCAACGCCAGCCAGGGCTTCAAGCGCTGGGAGTGCAACCGGCCGCACGCCCCGCACAGCCCCATCAAGTTCTCGGAGAAGTTCCAGCGCTACAGCGCCTTCTCTCTGGGCTACGAGTTCCACGCCGGCCACGAGTACTACTACATCTCCACGCCCACTCACAACCTGCACTGGAAGTGTCTGAGGATGAAGGTGTTCGTCTGCTGCGCCTCCACATCGCACTCCGGGAGAAGCCGGTCCCCACTCTCCCCCAGTTCACCATGGGCCCCAATGTGAAGATCAACGTGCTGGAAGACTTTGAGGGAGAGAACCCTCAGGTGCCAAGCTTGAGAAGAGCATCAGCGGGACCAGCCCCAAACGGGAACACCTCCCCTGGCCGTGGGCATCGCCTTCTTCCTCATGACGTTCTTGGCCTCCTAG) and pcDNA 3.1 vector served as the NC were purchased from HANBIO (Shanghai, China), and pcDNA3.1-EFNA3 was used to overexpressed EFNA3. Cells were seeded at 2 × 10^5^ cells/well in six-well plates overnight and then transfected by Lipofectamine 2000 reagent with plasmid. After transfections for 48 h, the expression of EFNA3 was evaluated by PCR and Western blot.

### CCK8 assay and colony-forming experiments

Cells (1500 cells/well) were cultured in 96-well plates and transfected with siRNA or Plasmid. After culture for 24, 48, or 72 h, cells were cultured with the CCK8 solution (C0038, Beyotime, Shanghai, China) for an additional 2 h. Cell viability was expressed as an optical density (OD) value at 450 nm. In order to examine the effects of EFNA3 expression on human LUAD cell proliferation, A549 cells (500/well) transfected with NC-siRNA or siRNA were added to the 12-well plates. After 10 days, the number of colonies were counted.

### Analysis of lactate, ATP, and glucose uptake levels

Cells (2 × 10^5^ cells/well) were cultured in 6-well plates and transfected with siRNA or Plasmid. The culture medium and cells were collected after 48 h. Lactate levels in the medium were determined with use of the lactate assay kit (ab65331, Abcam), ATP levels with the ATP assay kit (ab83355, Abcam) and glucose uptake levels with the glucose uptake assay kit (ab136955, Abcam). All determinations were normalized with cell numbers.

### Tumor infiltrating immune analysis

Estimate scores, stromal scores, and immune scores were obtained via ESTIMATE, which is a method that uses gene expression signatures to infer the fraction of stromal and immune cells. And, TISIDB database was used to analyze the relationships between levels of EFNA3 expression and lymphocyte, which integrates 988 immune-associated anti-tumor genes, high-throughput screening techniques, molecular profiles, and paracancerous multi-omics data, as well as various immunological data developed from 7 public databases.

### Immunotherapy response biomarkers

Immunotherapy response biomarker scores of patients with LUAD from the TCGA dataset were obtained from TCIA database (The Cancer Immunome Atlas, https://tcia.at/home) and TIDE database (Tumour Immune Dysfunction and Exclusion, http://tide.dfci.harvard.edu). Specifically, the mutation burden, number of neoantigens, number of clonal neoantigens, and number of subclonal neoantigens in patients with LUAD, obtained from the TCGA dataset, were obtained through TCIA database [20]. The TIDE score, T cell dysfunction score, and T cell exclusion score of patients with LUAD from the TCGA dataset were downloaded from the TIDE database. TIDE is a computational framework construct by Jiang et al. [21] to predict immune checkpoint blockade response. The TIDE signature was validated and outperformed known immunotherapy biomarkers that could predict immunotherapy response in melanoma and lung cancer, especially in patients treated with anti-CTLA4 and anti-PD-1/PDL1 [21].

### Statistical analysis

R (4.0) software was used for statistical analyses. Statistical comparisons were calculated using ANOVA tests, p-values < 0.05 were considered statistically significant.

## Results

### mRNA expression level of Eph/Ephrins in LUAD based on different databases

**Fig. 1:**
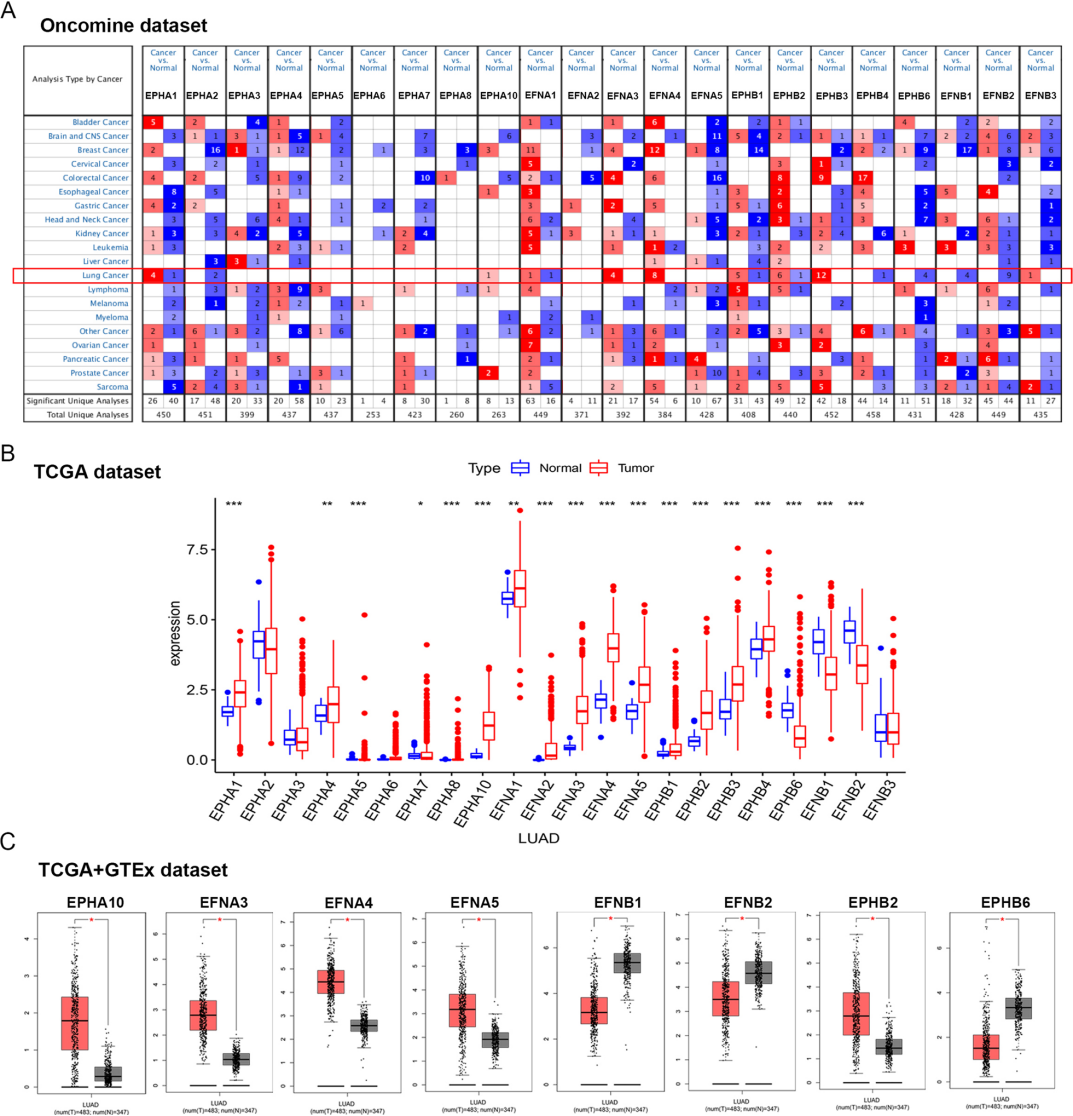
Expression level of Eph/Ephrins in LUAD based on different databases (**A**). The expression level of Eph/Ephrins in different types cancer based on Oncomine database; The color intensity (red or blue) is directly proportional to the significance level of upregulation or downregulation, respectively; **B** The expression level of Eph/Ephrins in LUAD based on TCGA dataset; **C** The expression level of Eph/Ephrins in LUAD based on TCGA and GTEx database, red plot and gray plot represent LUAD tissue and normal lung tissue respectively. *P < 0.05, **P < 0.01 and ***P < 0.001

First, the Oncomine database was used to analyze the mRNA expression levels of Eph/Ephrins in LUAD (Fig. 1A; Table 1). The following thresholds were used to analyze the data: twofold change, P value < 0.0001, and a gene grade of 10%.

**Table 1:** Datasets of Eph/Ephrins in lung cancer (Oncomine)

Gene	Tumor (cases)	Normal (cases)	Fold change	P-value	Dataset
EPHA1	Lung Adenocarcinoma (86)	Lung (10)	1.716	5.67E−10	Beer et al
	Lung Adenocarcinoma (58)	Lung (58)	1.702	1.78 E−14	Selamat et al
	Squamous Cell Lung Carcinoma (13)	Lung (6)	1.616	0.002	Garber et al
	Squamous Cell Lung Carcinoma (27)	Lung (65)	1.878	2.14 E−8	Hou et al
	Small Cell Lung Carcinoma (6)	Lung (17)	− 4.424	3.63 E−5	Bhattacharjee et al
EPHA2	Large Cell Lung Carcinoma (19)	Lung (65)	− 3.583	7.45 E−13	Hou et al
	Small Cell Lung Carcinoma (4)	Lung (6)	− 2.827	0.009	Garber et al
EPHA10	Lung Adenocarcinoma (226)	Lung (20)	4.732	5.65 E−10	Okayama et al
EFNA1	Squamous Cell Lung Carcinoma (34)	Lung (2); Tongue (26)	2.181	3.32 E−10	Talbot et al
	Lung Carcinoid Tumor (20)	Lung (17)	− 65.441	5.13 E−9	Bhattacharjee et al
EFNA3	Lung Adenocarcinoma (27)	Lung (30)	4.604	2.17 E−8	Su et al
	Squamous Cell Lung Carcinoma (27)	Lung (65)	1.934	9.04 E−17	Hou et al
	Large Cell Lung Carcinoma (19)	Lung (65)	1.943	1.88 E−7	Hou et al
	Lung Adenocarcinoma (86)	Lung (10)	1.791	2.70 E−4	Beer et al
EFNA4	Lung Adenocarcinoma (45)	Lung (65)	2.504	1.35 E−17	Hou et al
	Squamous Cell Lung Carcinoma (27)	Lung (65)	2.20	1.04 E−12	Hou et al
	Lung Adenocarcinoma (58)	Lung (58)	1.722	1.27 E−21	Selamat et al
	Lung Adenocarcinoma (27)	Lung (30)	3.720	2.97 E−8	Su et al
	Lung Adenocarcinoma (58)	Lung (49)	2.137	2.98 E−17	Landi et al
	Squamous Cell Lung Carcinoma (5)	Lung (6)	1.858	3.72 E−4	Wachi et al
	Lung Adenocarcinoma (226)	Lung (20)	2.423	8.49 E−15	Okayama et al
	Lung Adenocarcinoma (20)	Lung (19)	2.723	1.27 E−5	Stearman et al
EPHB1	Large Cell Lung Carcinoma (4)	Lung (6)	2.833	0.001	Garber et al
	Lung Adenocarcinoma (36)	Lung (6)	2.608	1.01 E−4	Garber et al
	Squamous Cell Lung Carcinoma (12)	Lung (6)	3.360	1.35 E−4	Garber et al
	Small Cell Lung Carcinoma (4)	Lung (6)	4.877	0.010	Garber et al
	Squamous Cell Lung Carcinoma, Basaloid Variant (8)	Lung (390)	1.543	5.08 E−4	TCGA
	Lung Adenocarcinoma (38)	Lung (6)	− 2.966	4.77 E−6	Garber et al
EPHB2	Lung Adenocarcinoma (20)	Lung (19)	1.947	1.15 E−7	Stearman et al
	Lung Adenocarcinoma (27)	Lung (30)	2.958	7.60 E−7	Su et al
	Lung Adenocarcinoma (45)	Lung (65)	1.539	1.22 E−11	Hou et al
	Lung Adenocarcinoma (58)	Lung (49)	1.594	6.46 E−13	Landi et al
	Lung Adenocarcinoma (132)	Lung (17)	2.006	0.015	Bhattacharjee et al
	Lung Adenocarcinoma (226)	Lung (20)	2.400	1.02 E−10	Okayama et al
	Lung Adenocarcinoma (86)	Lung (10)	− 2.372	2.95 E−6	Beer et al
EPHB3	Squamous Cell Lung Carcinoma, Basaloid Variant (8)	Lung (390)	1.721	1.21 E−4	TCGA
	Squamous Cell Lung Carcinoma (348)	Lung (390)	1.560	1.29 E−87	TCGA
	Squamous Cell Lung Carcinoma (11)	Lung (3)	1.779	0.004	Yamagata et al
	Large Cell Lung Carcinoma (5)	Lung (3)	1.563	0.014	Yamagata et al
	Squamous Cell Lung Carcinoma (13)	Lung (6)	4.747	7.31 E−5	Garber et al
	Squamous Cell Lung Carcinoma (21)	Lung (17)	5.147	6.27 E−4	Bhattacharjee et al
	Squamous Cell Lung Carcinoma (5)	Lung (6)	1.573	0.001	Wachi et al
	Lung Adenocarcinoma (20)	Lung (19)	1.797	1.29 E−4	Stearman et al
	Squamous Cell Lung Carcinoma (27)	Lung (65)	2.130	5.56 E−10	Hou et al
	Lung Adenocarcinoma (45)	Lung (65)	1.554	4.02 E−9	Hou et al
	Large Cell Lung Carcinoma (19)	Lung (65)	1.899	3.11 E−5	Hou et al
	Lung Adenocarcinoma (226)	Lung (20)	1.731	1.24 E−8	Okayama et al
EPHB4	Small Cell Lung Carcinoma (6)	Lung (17)	− 6.614	3.49 E−6	Bhattacharjee et al
EPHB6	Large Cell Lung Carcinoma (3)	Lung (6)	− 1.542	0.003	Garber et al
	Lung Adenocarcinoma (39)	Lung (6)	− 1.708	4.32 E−4	Garber et al
	Small Cell Lung Carcinoma (6)	Lung (17)	− 4.770	3.28 E−5	Bhattacharjee et al
	Lung Adenocarcinoma (20)	Lung (19)	− 3.105	1.18 E−4	Stearman et al
EFNB1	Lung Adenocarcinoma (20)	Lung (19)	− 1.789	4.73 E−8	Stearman et al
	Lung Carcinoid Tumor (20)	Lung (17)	− 16.859	8.76 E−7	Bhattacharjee et al
	Small Cell Lung Carcinoma (6)	Lung (17)	− 3.585	8.46 E−5	Bhattacharjee et al
	Large Cell Lung Carcinoma (4)	Lung (6)	− 1.592	0.017	Garber et al
EFNB2	Lung Adenocarcinoma (132)	Lung (17)	− 3.676	1.56 E−6	Bhattacharjee et al
	Lung Carcinoid Tumor (20)	Lung (17)	− 9.762	6.10 E−10	Bhattacharjee et al
	Squamous Cell Lung Carcinoma (21)	Lung (17)	− 2.156	0.009	Bhattacharjee et al
	Lung Adenocarcinoma (20)	Lung (19)	− 2.453	2.77E−6	Stearman et al
	Lung Adenocarcinoma (58)	Lung (49)	− 2.304	5.93E−17	Landi et al
	Lung Adenocarcinoma (27)	Lung (30)	− 2.044	2.96E−6	Su et al
	Large Cell Lung Carcinoma (19)	Lung (65)	− 3.549	9.56 E−10	Hou et al
	Squamous Cell Lung Carcinoma (5)	Lung (5)	− 1.917	0.004	Wachi et al
	Lung Adenocarcinoma (58)	Lung (58)	− 1.988	2.48 E−10	Selamat et al
EFNB3	Lung Carcinoid Tumor (20)	Lung (17)	4.500	1.58 E−5	Bhattacharjee et al

Overall, in most datasets, *EPHA1*, *EPHA10, EFNA3, EFNA4*, *EPHB1*, *EPHB2*, *EPHB3*, and *EFNB3* were upregulated in LUAD tissue compared with normal lung tissue. *EPHA2*, *EPHB4*, *EPHB6*, and *EFNB1* were downregulated in LUAD tissue. To further evaluate Eph/Ephrins expression, The Cancer Genome Atlas (TCGA) was used.

Since there were fewer normal samples in the TCGA dataset, the GTEx dataset based on the GEPIA website was included for further analysis of the differential expression of Eph/Ephrins between normal and LUAD tissue (Fig. 1B). As shown in Fig. 1C, the results were consistent with data from the Oncomine database and TCGA database. The data are as follows: *EPHA10*, *EFNA3*, *EFNA4*, *EFNA5*, *EPHB1*, and *EPHB2* expression levels were significantly upregulated in LUAD. In addition, *EFNB1*, *EFNB2*, and *EPHB6* expression levels were significantly downregulated in LUAD. Based on these results, *EPHA10*, *EFNA3*, *EFNA4*, *EFNA5*, *EPHB1*, *EPHB2*, *EFNB1*, *EFNB2*, and *EPHB6* were used for the next analysis.

### EFNA3 acts as the most valuable prognostic biomarker in LUAD patients

**Fig. 2:**
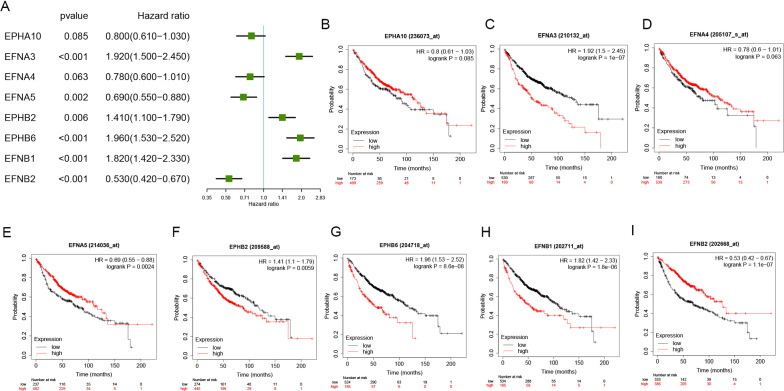
Prognostic values of Eph/Ephrins in LUAD (OS in Kaplan–Meier plotter). **A** Prognostic HR value of individual Eph/Ephrins in LUAD; **B**–**I** Prognostic significance of individual Eph/Ephrins in LUAD

**Fig. 3:**
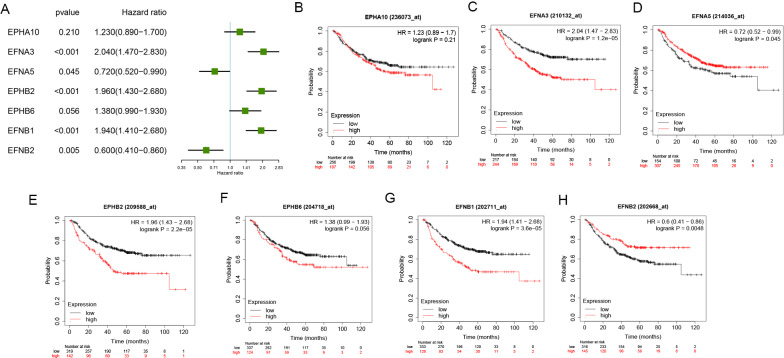
Prognostic values of Eph/Ephrins in LUAD (PFS in Kaplan–Meier plotter). **A** Prognostic HR value of individual Eph/Ephrins in LUAD; **B**–**H** Prognostic significance of individual Eph/Ephrins in LUAD

To evaluate the prognostic value of the selected Eph/Ephrins in LUAD patients, the Kaplan–Meier plotter database was used to analyze the relationship between expression levels and OS or PFS (Figs. 2, 3). First, the relationship with OS was analyzed. In Fig. 2A–I, upregulation of *EFNA3*, *EFNB2*, *EFNB1*, and *EPHB6* expression showed a significant correlation with poor OS in LUAD patients. In contrast, *EFNA5* and *EFNB2* upregulation signified a better prognosis. Conversely, *EPHA10* and *EFNA4* expression did not show a significant correlation with OS, so they were excluded from this study.

Next, *EFNA3*, *EFNB2*, *EFNB1*, *EPHB6*, *EFNA5*, and *EFNB2* were selected for correlation of the relationship between expression levels and PFS. As shown in Fig. 3A–H, upregulation of *EFNA3*, *EFNB1*, and *EPHB2* expression showed a significant correlation with poor PFS in LUAD patients. In contrast, *EFNB2* upregulation signified a better prognosis. Similarly, *EPHA10*, *EFNA5*, and *EPHB6* expression did not show a significant correlation with PFS.

**Fig. 4:**
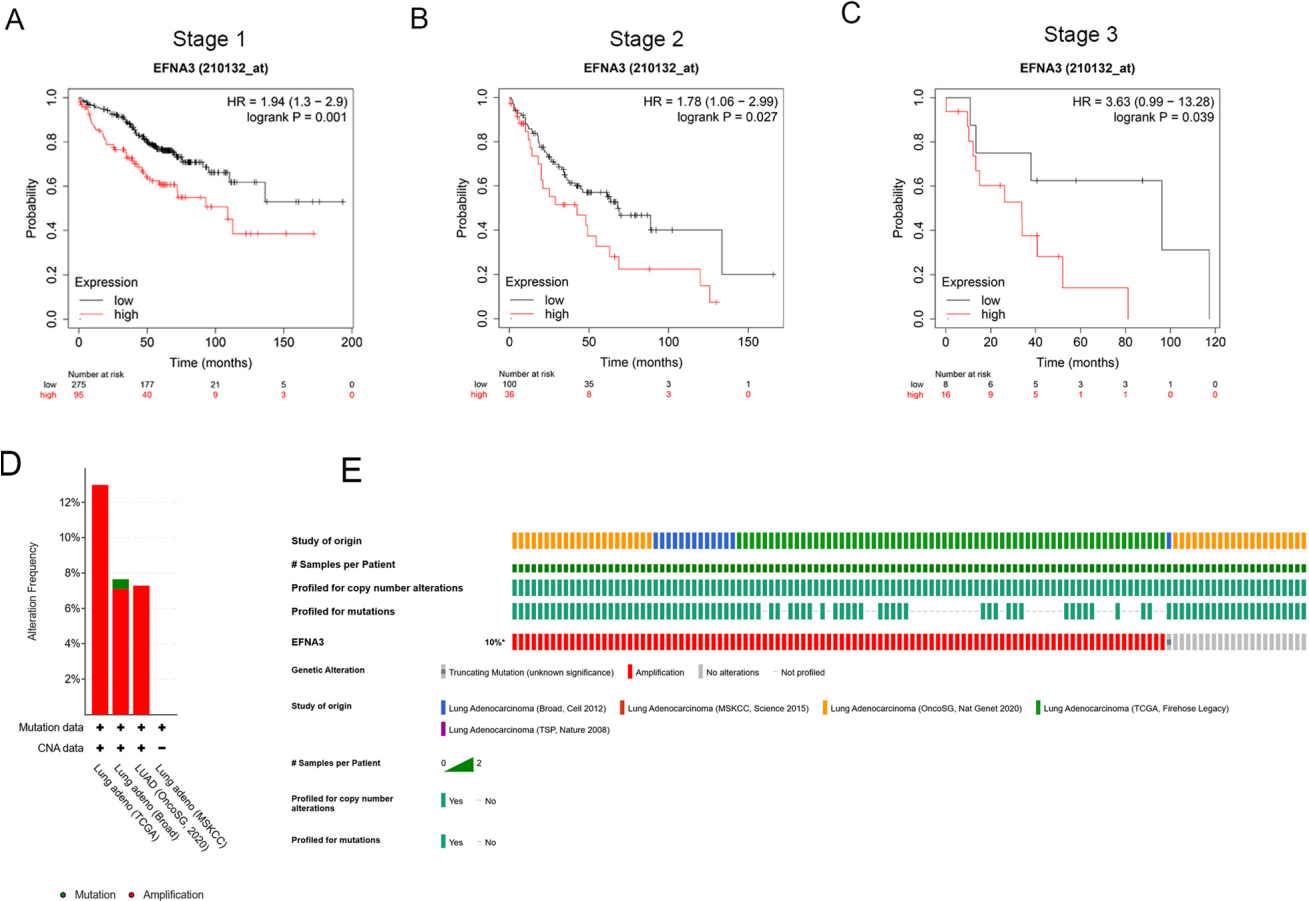
Survival analyses and genetic variations of EFNA3 in LUAD. **A**–**C** Prognostic significance of EFNA3 expression in differential stage LUAD patients. **D** Genetic variations in the EFNA3 gene reported in different studies; **E** OncoPrint overview of the genetic variations in the EFNA3 gene

*EFNA3* was selected next since it showed the highest HR value both in prognostic analysis for OS and PFS in LUAD patients*.* Patients with different stages of cancers require different therapeutic strategies and have different prognoses; therefore, subgroup analysis was applied[22]. As shown in Fig. 4A–C, higher *EFNA3* expression was associated with significantly worse OS, regardless of whether the patient was diagnosed at stage 1 (Fig. 4A), stage 2 (Fig. 4B) or stage 3 (Fig. 4C).

### Genetic variations of EFNA3

To determine whether upregulation of *EFNA3* in LUAD tissues was caused by genetic variations, genetic variations of *EFNA3* were assessed using the cBioPortal database. This database contains information on 1272 samples from five studies (Broad, Cell 2012 [23]; MSKCC, Science 2015 [24]; OncoSG, Nat Genet 2020 [25]; TCGA, Firehose Legacy [26]; TSP, Nature 2008 [27]). Genetic variations in *EFNA3* showed incidence rates of 12.98% in TCGA, 7.65% in Broad, and 7.28% in OncoSG (Fig. 4D, E). Amplification was the most common type (67/67 in TCGA; 1/14 in Broad; and 7/7 in OncoSG). Based on these results, amplification might be one of the main mechanisms by which *EFNA3* is highly expressed in LUAD.

### The expression of the EFNA3 protein was increased in LUAD tissues and related to the prognosis of LUAD patients

**Fig. 5:**
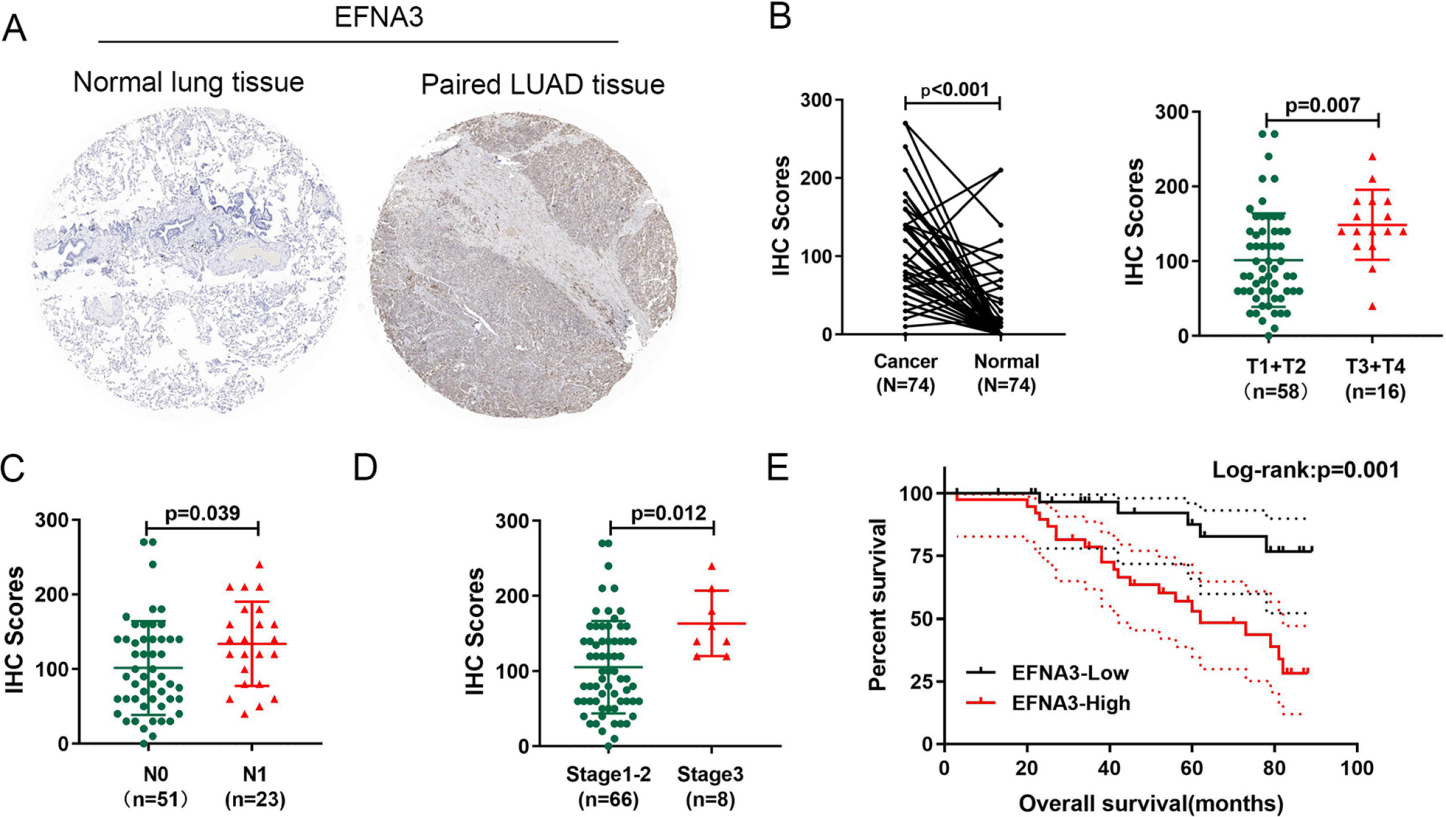
Upregulation of EFNA3 in lung adenocarcinoma (LUAD) tissues and its relationship with clinicopathological features and outcomes. **A** Representative images of EFNA3 staining in LUAD tissue and paired normal lung tissue; **B** The IHC score of EFNA3 was significantly increased in LUAD tissues; EFNA3 staining score increased significantly with tumor size (**C**), lymph node metastasis (**D**) and advanced TNM stages (**E**); **F** Kaplan–Meier survival analysis and log-rank tests showed that high expression levels of EFNA3 were associated with poor prognosis (P = 0.001)

To better understand the clinical significance of the EFNA3 protein in LUAD patients, IHC was performed to investigate the expression of EFNA3 in a tissue microarray (TMA) containing 74 LUAD tissues and adjacent normal lung tissues (Fig. 5A). Compared with normal lung tissue, the expression of the EFNA3 protein was significantly increased in LUAD tissue samples (Fig. 5B). Further analysis revealed that the IHC score for EFNA3 was significantly upregulated in cases of larger tumor sizes (Fig. 5C), lymph node metastasis (Fig. 5D), and advanced TNM stage (Fig. 5E).

**Fig. 6:**
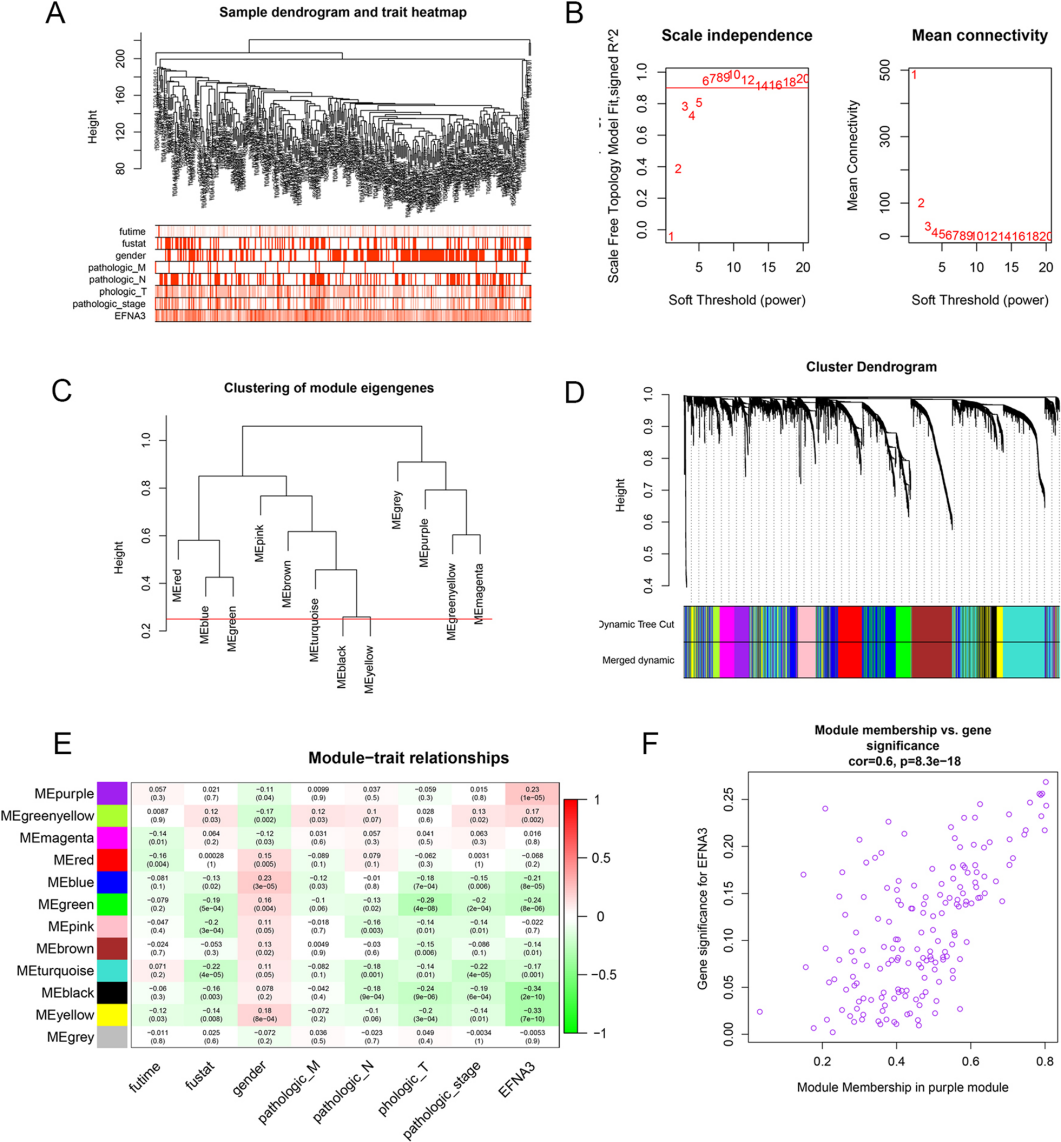
Screening for key modules related to EFNA3 through WGCNA. **A** Sample dendrogram and trait heatmap (TCGA-LUAD); **B** Calculation of the scale-free fit index of various soft-thresholding powers (β); **C** Clustering of module eigengenes. The red line indicates cut height (0.25); **D** Clustering dendrograms; **E** Correlation heatmap between module eigengenes and clinical parameters; **F** Scatter plot of purple module eigengenes

LUAD patients were divided into two groups according to IHC scores: the EFNA3-high group and the EFNA3-low group. The chi-square test showed that EFNA3 protein expression was significantly correlated with larger tumor size (P = 0.004), lymph node metastasis (P = 0.035), and a higher TNM stage (P = 0.002) (Table 2). Moreover, EFNA3-negative patients presented with shorter overall survival times than EFNA3-positive patients (P = 0.039) (Fig. 6F). Finally, Cox regression analysis of overall survival showed that higher EFNA3 expression was an independent prognostic risk factor (HR = 3.108; 95% CI = 1.077–8.963; P = 0.036) (Table 3). Based on these results, EFNA3 could represent a new prognostic biomarker for LUAD.

**Table 2:** Correlation between the expression of EFNA3 and clinical characteristics in LUAD patients (n = 74)

Clinical Pathological	Number	EFNA3 expression		P value
Parameters		High(n = 38)	Low(n = 36)	
Age				0.367
< 60	29	13	16	
≥ 60	45	25	20	
Sex				0.252
Male	48	27	21	
Female	26	11	15	
Tumor size				0.004*
T1	15	5	10	
T2	43	19	24	
T3 + T4	16	14	2	
LN metastasis				0.035*
No	51	22	29	
Yes	23	16	7	
TNM stage				0.002*
I	31	10	21	
II	35	20	15	
III + IV	8	8	0	

^*^Significant correlation

**Table 3:** Cox regression analysis of overall survival in LUAD patients

Variables	Univariate analysis			Multivariate analysis		
	HR	95% CI	P value	HR	95% CI	P value
Age (years) (≤ 60 vs > 60)	1.180	0.529–2.629	0.686			
Gender (male vs female)	0.949	0.419–2.150	0.900			
pT stage	1.489	0.898–2.469	0.123			
pN stage	3.162	1.435–6.965	0.004*	1.336	0.479–3.725	0.580
pTNM stage	2.583	1.505–4.432	<0.001*	1.530	0.738–3.173	0.253
EFNA3 expression	4.272	1.597–11.423	0.004*	3.108	1.077–8.963	0.036*

Factors for which P < 0.05 in univariate analysis were subsequently used for multivariate analysis

### WGCNA and GSEA

**Fig. 7:**
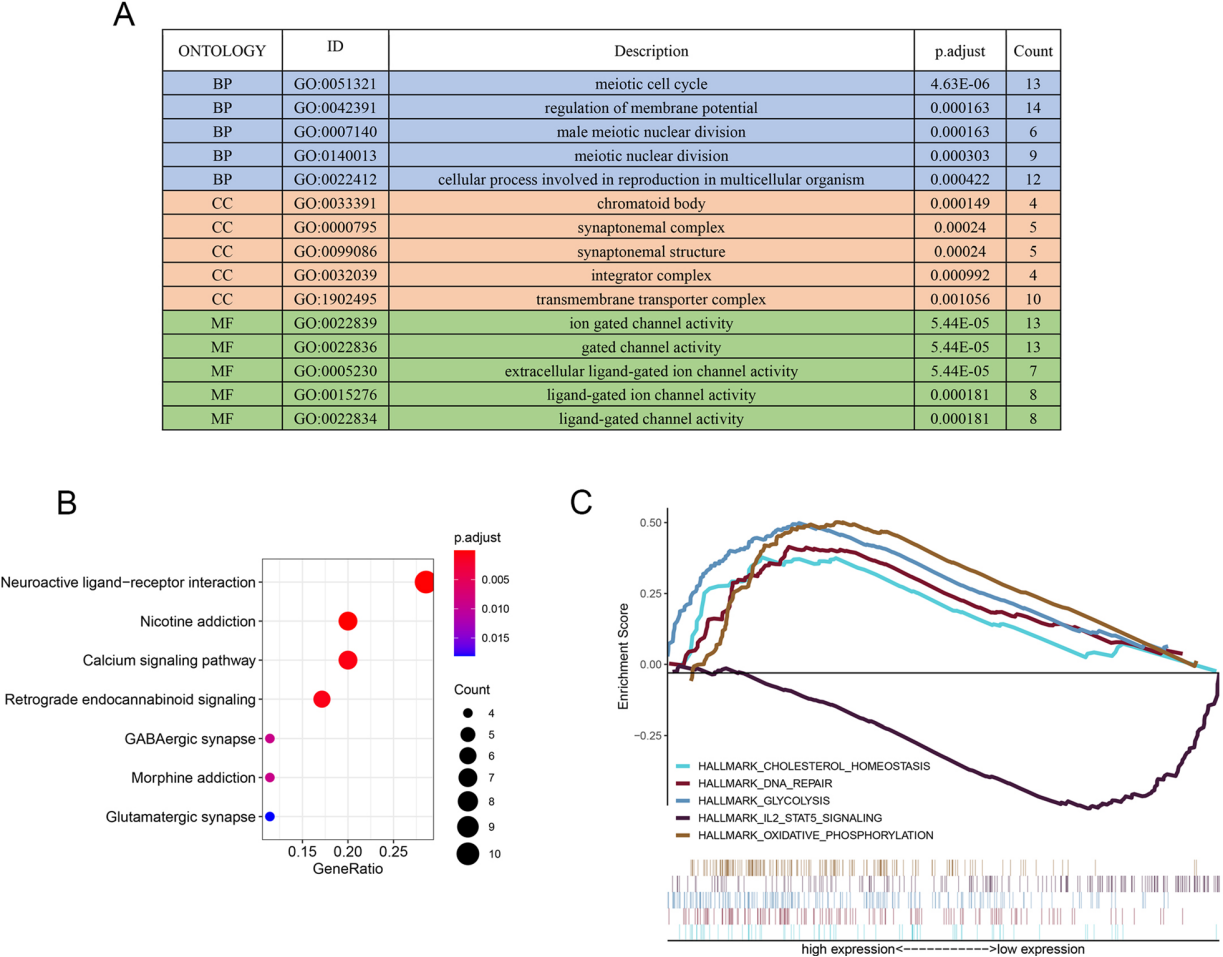
Potential regulate mechanisms of EFNA3 in LUAD. **A** GO analysis of 168 purple module eigengenes; BP: biological processes; CC: cellular components; MF: molecular functions; **B** KEGG analysis of purple module eigengenes; **C** Enriched pathways in the EFNA3-high and EFNA3-low group based on GSEA analysis

To identify the potential regulatory mechanism of *EFNA3,* the TCGA dataset was used to construct the coexpression network through WGCNA. Clinical features, including OS time, OS status, pathological parameters and *EFNA3* expression, were obtained from the TCGA dataset (Fig. 6A). The parameters were established by setting the soft-threshold power to 3 (scale free R2 = 0.0.868), and the height was set to 0.25. In this study, 12 modules were identified (Fig. 6B–D). The association between the modules and clinical features was measured by the correlation between module eigengene (ME) values and clinical features. Data were visualized by heatmap profiles. The results showed that the purple module was the most closely correlated with *EFNA3* expression (Pearson coefficient = 0.23, P = 1E−05; Fig. 6E). A scatter plot of purple module eigengenes is shown in Fig. 7F. In the purple module, 168 genes were selected as hub genes for GO and KEGG analysis.

In the purple module genes, nuclear division, synaptic function, and ion channel activity-related pathways were the most frequently noted pathways in the GO analysis. The most enriched GO term in the biological process (BP) category was “meiotic cell cycle.” In the cellular component (CC) category, “chromatoid body” was most enriched, and in the molecular function (MF) category, “ion gated channel activity” was most abundant. Furthermore, KEGG pathway enrichment analysis results contained "neuroactive ligand-receptor interaction" and "calcium signaling pathway" enrichments (Fig. 7B). Finally, GSEA was used to identify the mechanism and functional differences between the *EFNA3*-high expression group and the *EFNA3*-low expression group. As shown in Fig. 7C, "cholesterol homeostasis," "DNA repair," "glycolysis," and “oxidative phosphorylation" were enriched in the *EFNA3*-high expression group, whereas "IL2-STAT5 signaling" was enriched in the *EFNA3*-low expression group.

### The function of EFNA3 and its regulation of glycolysis ability

**Fig. 8:**
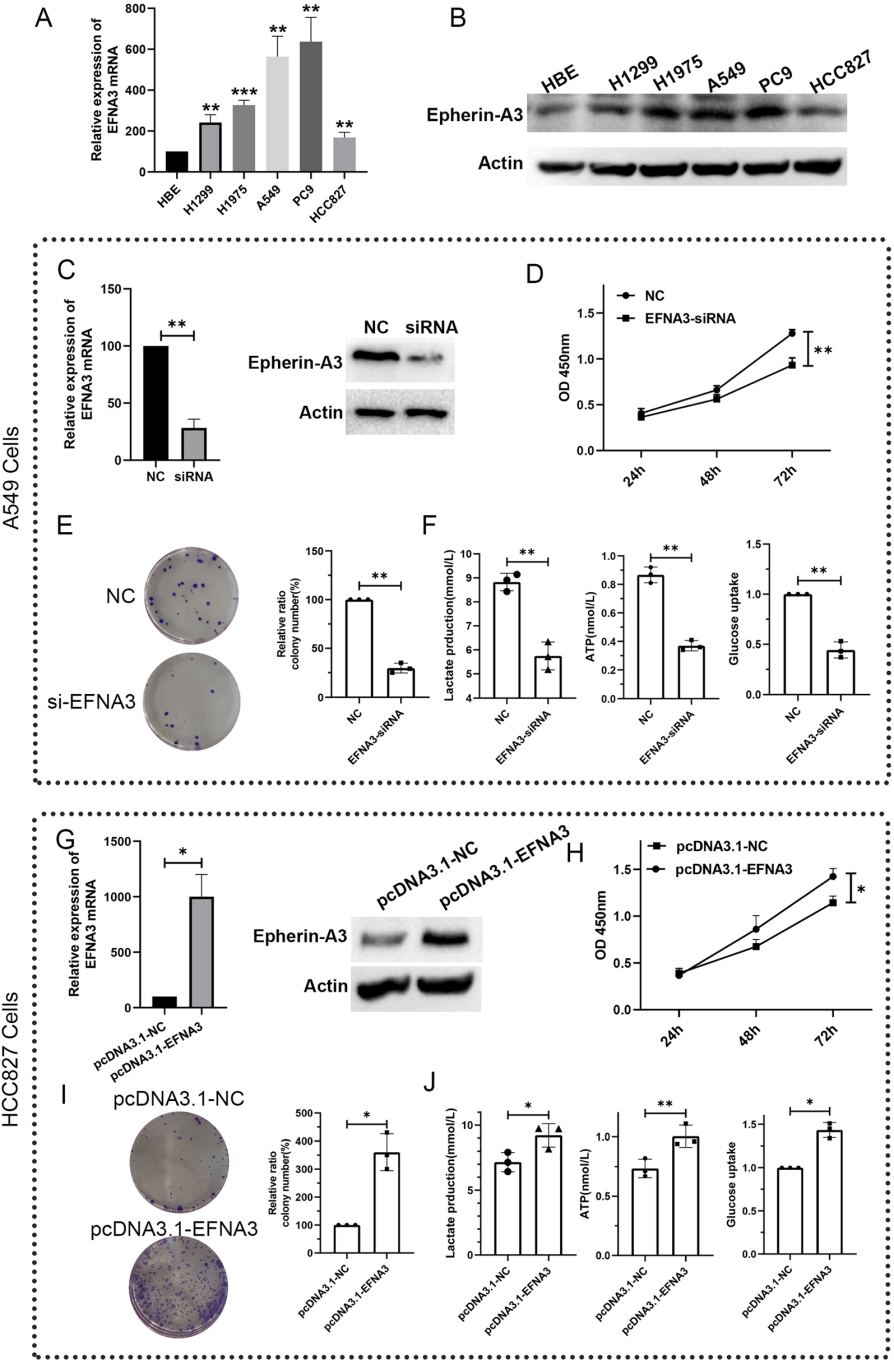
EFNA3 regulate the proliferation ability and glycolytic capacity of A549 cells. mRNA (**A**) and protein (**B**) expression levels of EFNA3 in human bronchial epithelial cells (HBE) and lung adenocarcinoma cells (H1299, H1975, A549, PC9, and HCC827). **C** EFNA3 expression levels were detected by qRT-PCR (left) and western blotting (right) analyses after transfection with negative control (NC) or EFNA3-siRNA; **D** Cell viability assays showed that EFNA3 knockdown decreased A549 cell proliferation; **E** The number of colonies formed by A549 cells transfected with EFNA3‐siRNA was lower than that for cells transfected with NC. **F** Lactate production, ATP level, and glucose uptake were measured in A549 cells after transfection with negative control (NC) or EFNA3-siRNA. **G** EFNA3 expression levels were detected by qRT-PCR (left) and western blotting (right) analyses after transfection with negative control (pcDNA3.1-NC) or pcDNA3.1-EFNA3; **H** Cell viability assays showed that EFNA3 overexpression increased HCC827 cell proliferation; **I** The number of colonies formed by HCC827 cells transfected with pcDNA3.1-EFNA3was higher than that for cells transfected with pcDNA3.1-NC; **J** Lactate production, ATP level, and glucose uptake were measured in HCC827 cells after transfection with negative control (pcDNA3.1-NC) or pcDNA3.1-EFNA3. mean ± SD; *p < 0.05, **p < 0.01, and ***p < 0.001 (vs. control group)

Sustained proliferation is the most fundamental feature of cancer cells. To investigate whether EFNA3 can regulate cell proliferation ability in LUAD, we first detected the mRNA and protein expression levels of EFNA3 in human bronchial epithelial cells and lung adenocarcinoma cells. As seen in Fig. 8A, B, the mRNA and protein expression levels of EFNA3 were upregulated in LUAD cells (H1299, H1975, A549, PC9, and HCC827) compared to HBE cells. A549 cells were chosen for the next study for further investigation because of their high levels of EFNA3 expression. Knockdown of EFNA3 expression was performed using siRNA. Transfection efficiency was measured using qRT–PCR and western blot analyses (Fig. 8C). CCK-8 assays revealed that knockdown of EFNA3 significantly decreased the proliferation of A549 cells (Fig. 8D). Moreover, the EFNA3-siRNA-transfected group had significantly fewer colonies than the NC-siRNA-transfected group (Fig. 8E). Taken together, these results demonstrated that EFNA3 knockdown could decrease the proliferation ability of LUAD cells in vitro. In Fig. 8C, we found that the glycolysis pathway was enriched in the EFNA3-high expression group. Therefore, we next explored whether EFNA3 is involved in regulating the glycolytic ability of LUAD cells. Functional colorimetric validation (Fig. 8F) showed that lactic acid production (a key metabolite of glycolysis), ATP levels and glucose uptake were both significantly decreased after EFNA3 knockdown in A549 cells.

To further validate these results, we next overexpressed EFNA3 in the HCC827 cell line, which normally expresses a low level of EFNA3. Transfection efficiency was quantified by qRT–PCR and western blotting (Fig. 8G). CCK-8 assays revealed that overexpression of EFNA3 significantly upregulated the proliferation of HCC827 cells (Fig. 8H). Moreover, the pcDNA3.1-EFNA3-transfected group had significantly more colonies than the pcDNA3.1-NC-transfected group (Fig. 8I). Finally, lactic acid production, ATP levels and glucose uptake were all significantly increased after EFNA3 overexpression in HCC827 cells. These results suggest that EFNA3 could regulate the glycolytic capacity of LUAD cells.

### Correlation analysis between EFNA3 expression and microenvironment

**Fig. 9:**
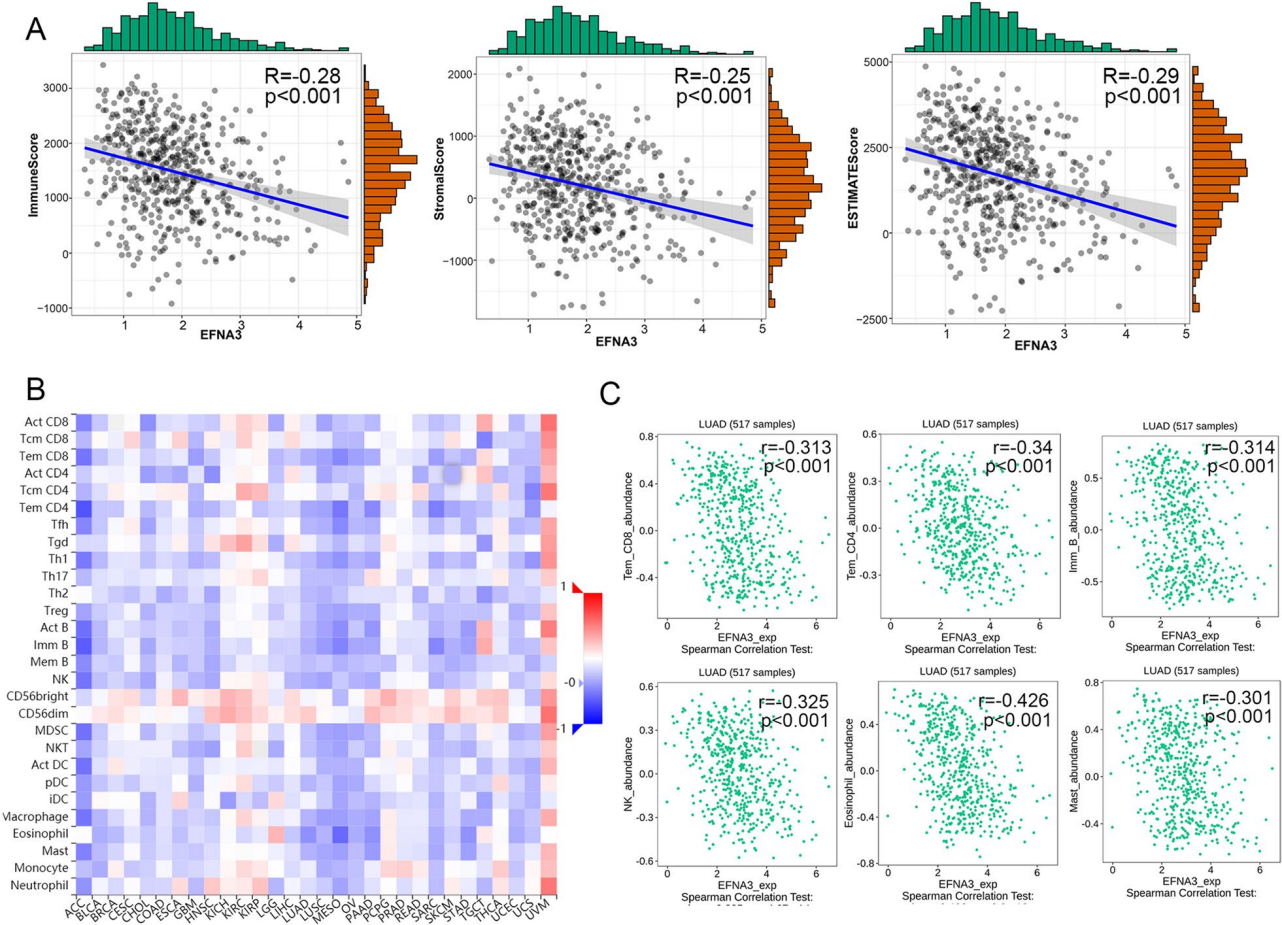
Correlation analysis between EFNA3 expression and immune infiltration. **A** The relationship between EFNA3 expression and immune scores, stromal scores, and ESTIMATE score; **B** The abundances of TILs correlated with EFNA3 expression. **C** Effector memory CD4 T cell (rho = − 0.34, p = 2.64e−15), effector memory CD8 T cell (rho = − 0.313, p = 4.6e−13), Immature B cell (Imm B cell) (rho = − 0.314, p = 3.58e−13), NK cell (rho = − 0.325, p = 4.67e−14), Eosinophil cell (rho = − 0.426, p = 2.2e−16), and mast cell (rho = − 0.301, p = 3.45e−12) was correlate with EFNA3 expression level in LUAD

Next, we assessed the correlation between EFNA3 expression and the tumor immune microenvironment. As shown in Fig. 9A, the expression of EFNA3 was negatively correlated with immune scores (R =  − 0.28, p < 0.001), stromal scores (R = − 0.25, p < 0.001) and ESTIMATE scores (R = − 0.29, p < 0.001). The relationships between the expression of EFNA3 and tumor-infiltrating lymphocytes (TILs) were analyzed by Spearman correlation using the TISIDB database (Fig. 9B). The results indicated a negative correlation between most TILs and EFNA3 levels. As shown in Fig. 9C, the TILs display moderate correlation(R < − 0.3) including effector memory CD4 T cell (rho = − 0.34, p = 2.64e−15), effector memory CD8 T cell (rho = − 0.313, p = 4.6e−13), Immature B cell (Imm B cell) (rho = − 0.314, p = 3.58e−13), NK cell (rho = − 0.325, p = 4.67e−14), Eosinophil cell (rho = − 0.426, p = 2.2e−16), and mast cell (rho = − 0.301, p = 3.45e−12).

### Association of EFNA3 and immunotherapy response in patients with LUAD

**Fig. 10:**
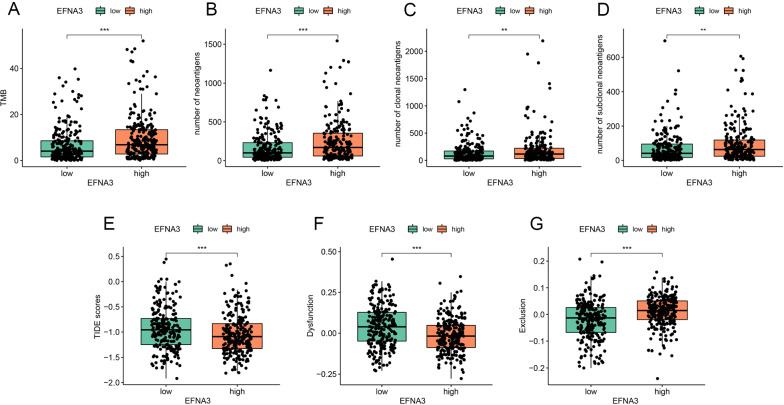
The expression pattern of immunotherapy response makers in high- and low-EFNA3 groups. The distribution of TMB (**A**), number of neoantigens (**B**), number of clonal neoantigens (**C**), number of subclonal neoantigens (**D**), TIDE score (**E**), T cell dysfunction score (**F**), T cell exclusion score (**G**) in EFNA3-high and EFNA3-low groups; *p < 0.05, **p < 0.01, and ***p < 0.001 (vs. control group)

Presently, immunotherapy is considered an important treatment for patients with LUAD. Therefore, we further assessed the association of EFNA3 and immunotherapy response by analyzing the correlation of EFNA3 expression and widely recognized immunotherapy biomarkers. In total, we enrolled seven indices, including TMB, the number of neoantigens, the number of clonal neoantigens, the number of subclonal neoantigens, the TIDE score, the T cell dysfunction score, and the T cell exclusion score. As depicted in Fig. 10A–H, patients in the EFNA3-high group were distinguished by a high level of TMB, neoantigens, and T cell exclusion scores and low levels of TIDE and T cell dysfunction scores. These results indicate that patients with higher expression of EFNA3 may benefit from immunotherapy.

## Discussion

Our research group aimed to study correlations between Eph/Ephrin family members and lung cancer. In this study, EFNA3, EFNB1, EFNB2, and EPHB2 showed a significant correlation with OS and PFS in LUAD patients and were differentially expressed in tumor tissues and normal lung tissues. EPHB2, as an EPHB subgroup receptor kinase, could modulate the biological behavior of small cell lung carcinoma through autocrine and/or juxtracrine activation by ephrin-B ligands (EFNB1, EFNB2, and EFNB3) that are expressed in the same or neighboring cells [28]. In addition, Zhao et al. reported that high expression of EPHB2 can predict poor overall survival and a high mortality rate and that it is an independent prognostic biomarker in lung adenocarcinoma patients [29]. Recent data have suggested that curcumin could regulate EPHB2 by suppressing Sp-1 activation [30]. The gene expression of EFNA3 was upregulated in early-stage NSCLC [31]. Recent data [32] suggested that EFNA3 was selectively downregulated in tumor samples of lung cancer patients with COPD due to higher expression of miR-210. Therefore, we believe that EFNA3, EFNB1, EFNB2, and EPHB2 are closely associated with the tumorigenesis and progression of LUAD.

The present study further focused on *EFNA3*, which had both significant differential expression and the most significant prognostic value. Studies have found that the expression levels of *EFNA3* are inhibited in the process of skeletal muscle satellite cell formation [33]. Another study showed that *EFNA3* promotes the proliferation and invasion of peripheral nerve sheath tumor cells and is regulated by miR-210 [34]. Importantly, several studies have shown that *EFNA3* is involved in tumor angiogenesis [34, 35]. However, the functions of *EFNA3* in the pathogenesis and progression of LUAD are still unclear. The results from the cBioPortal database suggest that amplification may be one of the main mechanisms by which *EFNA3* is overexpressed in LUAD. In this study, the expression level and prognostic value were further verified in LUAD through clinical samples. The protein expression of EFNA3 was upregulated in LUAD tissue compared to normal lung tissue. In addition, the expression of the EFNA3 protein was significantly related to clinicopathological characteristics. Thus, *EFNA3* gene expression is an independent prognostic risk biomarker.

According to previous studies, overexpression of EFNA3 can reduce glutamate transporter levels in astrocytes, and EphA4/EFNA3 signaling can regulate synaptic function and plasticity [36]. In addition, it has been confirmed that *EFNA3* could regulate the EMT process by the PI3K/AKT signaling pathway in oral cancer [37]. In this study, purple modules related to *EFNA3* were screened out through WGCNA. Further enrichment analysis showed that *EFNA3* is closely related to nuclear division, synaptic function, and ion channel activity. In addition, GSEA showed that "cholesterol homeostasis," "glycolysis," and "oxidative phosphorylation" were enriched in the *EFNA3*-high expression group, suggesting that *EFNA3* may be closely related to the metabolic ability of lung adenocarcinoma cells. In short, *EFNA3* may promote the malignant progression of LUAD through these potential pathways. Further experiments showed that EFNA3 knockdown could decrease the proliferation ability and glycolytic capacity of LUAD cells in vitro. Our research results suggest a potential oncogenic effect of EFNA3 in LUAD, but further research is needed to provide conclusive results. in particular, the specific oncogenic mechanism still needs further exploration.

In recent years, immunotherapy involving checkpoint inhibitors of the PD1/PDL1 axis [38, 39] has been used in clinical therapy for LUAD patients. In this study, we found that TMB, neoantigens, and TIDE scores were associated with patients in the EFNA3-high group. TMB is one of the classic biomarkers for immunotherapy response, and neoantigen burden is always increased by TMB [24, 40]. The TIDE score is a newly developed method for immunotherapy response prediction and is considered a more accurate biomarker than TMB [21]. Collectively, we preliminarily speculate that EFNA3-high patients may be suitable for immunotherapy. This might be due to ephrin ligands controlling cell interactions during normal development and playing a vital role in both locally and systemically induced immune responses [41, 42]. Importantly, we found that EFNA3 was associated with the tumor microenvironment and lymphocytes. These findings give us additional confidence that EFNA3 expression may act as a novel predictive biomarker for immunotherapy response. Prospective studies should be used to further determine the relationship between EFNA3 and immunotherapy response in the future.

Future research should focus on the regulatory mechanism of high EFNA3 expression in LUAD tissues. Several studies have confirmed that posttranscriptional modification plays an important role in regulating protein expression [43, 44]. MicroRNAs (miRNAs) are a family of posttranscriptional gene repressors and have been widely associated with the regulation of gene expression in various contexts [45]. Previous studies indicated that miR-210 could regulate the expression of EFNA3 by binding to its 3’-UTR in pancreatic cancer cells [46], peripheral nerve sheath tumor cells [47], oral cancer cells [37], etc. Moreover, one study demonstrated that miR-210 can regulate the expression of EFNA3 to promote sensory axon regeneration in adult mice [48]. Importantly, several studies indicate that miRNA-210 from extracellular vesicles could regulate the expression of EFNA3 to promote angiogenesis in ischemic hearts [49], oral squamous cell carcinoma [34], acute myocardial infarction [49], and ischemic disease models [50]. These studies suggest that abnormal expression of miR-210 is a possible cause of high EFNA3 expression in LUAD. Therefore, future experimental studies should be performed to explore the regulatory mechanism between miR-210 and EFNA3 in LUAD.

However, this study has some limitations. Our data indicate thatproveEFNA3 plays a role in promoting LUAD cells and could regulate glycolytic capacity, the underlying mechanism by which EFNA3 regulates glycolysis has not been elucidated. Second, because the mRNA expression data from patients with immunotherapy were not available, the prediction ability of EFNA3 for immunotherapy response was estimated indirectly. These experimental studies will be performed and reported in the future.

In conclusion, this study revealed that increased EFNA3 in LUAD patients predicted worse clinical prognosis, promoted LUAD cell proliferation and glycolysis ability, and was related to the immunotherapy response.

## Data Availability

Not applicable.
